# Poly(*N,N*-dimethylacrylamide)-coated upconverting NaYF_4_:Yb,Er@NaYF_4_:Nd core–shell nanoparticles for fluorescent labeling of carcinoma cells

**DOI:** 10.1038/s41598-021-00845-y

**Published:** 2021-11-01

**Authors:** Viktoriia Oleksa, Hana Macková, Hana Engstová, Vitalii Patsula, Oleksandr Shapoval, Nadiia Velychkivska, Petr Ježek, Daniel Horák

**Affiliations:** 1 Institute of Macromolecular Chemistry of the Czech Academy of Sciences, Heyrovského nám. 2, 162 06 Prague 6, Czech Republic; 2 Department of Physical and Macromolecular Chemistry, Faculty of Science, Charles University, Hlavova 8, 128 40 Prague 2, Czech Republic; 3 Institute of Physiology of the Czech Academy of Sciences, Vídeňská 1083, 142 20 Prague 4, Czech Republic

**Keywords:** Chemistry, Materials science

## Abstract

Upconverting luminescent lanthanide-doped nanoparticles (UCNP) belong to promising new materials that absorb infrared light able to penetrate in the deep tissue level, while emitting photons in the visible or ultraviolet region, which makes them favorable for bioimaging and cell labeling. Here, we have prepared upconverting NaYF_4_:Yb,Er@NaYF_4_:Nd core–shell nanoparticles, which were coated with copolymers of *N,N*-dimethylacrylamide (DMA) and 2-(acryloylamino)-2-methylpropane-1-sulfonic acid (AMPS) or *tert*-butyl [2-(acryloylamino)ethyl]carbamate (AEC-Boc) with negative or positive charges, respectively. The copolymers were synthesized by a reversible addition-fragmentation chain transfer (RAFT) polymerization, reaching *M*_n_ ~ 11 kDa and containing ~ 5 mol% of reactive groups. All copolymers contained bisphosphonate end-groups to be firmly anchored on the surface of NaYF_4_:Yb,Er@NaYF_4_:Nd core–shell nanoparticles. To compare properties of polymer coatings, poly(ethylene glycol)-coated and neat UCNP were used as a control. UCNP with various charges were then studied as labels of carcinoma cells, including human hepatocellular carcinoma HepG2, human cervical cancer HeLa, and rat insulinoma INS-1E cells. All the particles proved to be biocompatible (nontoxic); depending on their ξ-potential, the ability to penetrate the cells differed. This ability together with the upconversion luminescence are basic prerequisites for application of particles in photodynamic therapy (PDT) of various tumors, where emission of nanoparticles in visible light range at ~ 650 nm excites photosensitizer.

## Introduction

Although the first lanthanide-based upconverting luminescent materials were described already in the 1970s^1,2^ and upconverting nanoparticles (UCNP) were developed till in the late 1990s^3^, their thorough research began after 2004, when a number of publications started to increase rapidly^4–7^. Upconversion refers to a nonlinear optical process, where the sequential absorption of multiple low‐energy photons leads to emission of high‐energy photons through the long‐lived intermediate energy states^8^. However, these processes are limited by undesirable competing mechanisms, such as cross-relaxation, back-transfer, nonradiative decay, and energy transfer to high-energy vibrations on the particle surface^9^. Synthetic approaches overcoming these drawbacks consist in localization of activator and sensitizer ions in two different layers, passivation of the surface, selection of larger crystal lattices, or growing of an additional shell on the UCNP by the epitaxial layer-by-layer strategy. For example, core@multishell nanoparticles allowed generation of multicolor dual-modal luminescence applicable for anti-counterfeiting and/or construction of speed sensors^10,11^. The layer-by-layer technique is used also in this report to allow a more homogenous localization of activator ions in the crystalline lattice^12,13^.

UCNP typically consist of NaYF_4_ host lattice doped with an activator, e.g., Er^3+^, Tm^3+^, or Ho^3+^ ions (< 2 mol%), and a sensitizer, often Yb^3+^ ions (~ 20 mol%)^14^. Such dopant concentrations allow adequate distance between activator ions to avoid cross-relaxation and absorption of enough excitation radiation by the sensitizer to achieve a good efficiency. UCNP are typically synthesized by the precipitation of lanthanide salts, which results in polydisperse products with cubic α-phase structure, or thermal decomposition^15^ and hydro(solvo)thermal methods allowing to produce uniform nanoparticles with hexagonal β-phase^16^. Both crystalline phases provide different upconversion efficiency, with the cubic phase yielding usually the lower performance. Therefore, the research is mostly focused on tuning the physicochemical properties of UCNP and solving problems associated with quenching that lowers the upconversion efficiency^17^.

UCNP used for cell tracking and labeling compete with conventional water-soluble fluorescent markers developed for imaging of various cell organelles, specific proteins, or cell pathways^18–20^. Nevertheless, the advantage of UCNP consists in that they can be excited with near-infrared light (NIR) penetrating at 808 nm up to 5 cm deep into tissues, where it is upconverted to visible light^21^. This can be exploited for example in photodynamic therapy of tumors, the capillaries of which are typically fenestrated or immature enabling higher retention of photosenzitizers in the extracellular matrix. Another benefit of UCNP is the absence of photobleaching, spectrally distinct and narrow emissions, non-blinking, weak autofluorescence, high signal-to-noise ratio, and unique photostability. The weak point of nanoparticle-based labeling consists in the fact that the particles are often too large to readily diffuse across the negatively charged plasma cell membrane, which limits ability to interact with cognate intracellular targets. The mechanism of engulfment by cells is thus related to the size of nanoparticles and their aggregates. Involved can be pinocytosis (cell drinking), which is nonspecific, phagocytosis (cell eating) ingesting larger particles, or receptor-mediated endocytosis, when caveoline or clathrin proteins are engaged^22^. After the nanoparticle uptake, they may stay encapsulated by cell membranes in endosomes and lately in lysosomes, which protect them from reaching cytosol compartments. The nanoparticles are thus usually able to reach only the lysosomes, where they are trapped, without any chance to enter the inner cell environment. Moreover, majority of nanoparticles has only limited colloidal stability in the culture media due to electrostatic stabilization. In this regard, the steric stabilization by adsorbing or covalent binding hydrophilic polymers is preferred. Consequently, the nanoparticles are often decorated with charged molecules, such as saccharides, folic acid, various synthetic polymers, proteins, peptides^23,24^, or phospholipids^25^, to increase attractivity for cells. It is supposed that the larger and negatively charged particles are engulfed rather by phagocytic than nonphagocytic cells, which rather prefer smaller and positively charged nanoparticles^26,27^; however, also opposite results were published, e.g., for A549 cell line^28^. Bioapplications of positively charged particles (coated with polyethyleneimine, chitosan, polylysine, polyarginine, etc.) are also accompanied with higher toxicity due to disruption of plasma membrane integrity^26^, while negatively charged particles can induce intracellular damage^29^. Negatively charged polymers supporting cellular uptake of the nanoparticles are exemplified by polystyrene sulfonic acid^30^ and poly(γ-glutamic acid)^31^, while electroneutral polymers typically contain hydrophobic motives, e.g., esters of myristic acid^32^, or are based on silica. Polymer coatings for UCNP should also contain carboxyl, phosphate, (bis)phosphonate, or sulfate anchoring groups to facilitate attachment to the particle surface.

In this report, we investigated di-end-functionalized poly(*N,N*-dimethylacrylamide) copolymers containing both bisphosphonate anchoring groups binding to the surface of UCNP and amino or sulfonate groups supporting engulfment of the particles in human hepatocellular carcinoma HepG2, human cervical cancer HeLa, and rat insulinoma INS-1E cells. The ultimate goal was to design and develop new surface-engineered in vivo cell trackable UCNP excitable at 808 nm wavelength that excellently penetrate the tissues and are suitable for fluorescent labeling of carcinoma cells.

## Experimental

### Chemicals

Anhydrous lanthanide chlorides, i.e., yttrium(III), ytterbium(III), erbium(III), and neodymium(III) chloride (99%), ammonium fluoride (99.99%), octadec-1-ene (90%), *N,N*-dimethylacrylamide (DMA; 99%), 4,4′-azobis(4-cyanovaleric acid) (ACVA), 2-(dodecylthiocarbonothioylthio)-2-methylpropionic acid (DMP; chain transfer agent—CTA; 98%), 4-(dimethylamino)pyridine (DMAP; 99%), *N*,*N*′-dicyclohexylcarbodiimide (DCC; 99%), *N*-hydroxysuccinimide (NHS; 98%), 2-(acryloylamino)-2-methylpropane-1-sulfonic acid (AMPS), sodium borohydride (≥ 98%), 2-[4-(2-hydroxyethyl)piperazin-1-yl]ethanesulfonic acid (HEPES), and phosphate buffered saline (PBS) were purchased from Sigma-Aldrich (St. Luis, MO, USA). Oleic acid (OA; 98%) was purchased from Lachema (Brno, Czech Republic). Sodium salt of (4-amino-1-hydroxy-1-phosphonobutyl)phosphonic acid trihydrate (alendronate; Ale) was purchased from TCI (Tokyo, Japan). Fluorescent DY-615-maleimide dye was purchased from Dyomics (Jena, Germany). ɑ-Methoxy-ω-NHS poly(ethylene glycol) (NHS-PEG; *M*_w,PEG_ = 5000 Da) was obtained from Rapp Polymere (Tübingen, Germany) and absolute ethanol from LachNer (Neratovice, Czech Republic). CellMask™ green was purchased from Thermo Fisher Scientific (Waltham, MA, USA). *Tert*-butyl[2-(acryloylamino)ethyl]carbamate (AEC-Boc), poly(*N,N*-dimethylacrylamide) (PDMA), poly(*N,N*-dimethylacrylamide) (PDMA-Ale), sodium neridronate, and neridronate-PEG (Ner-PEG) were prepared as described in previous reports^33–36^. Cellulose dialysis membranes (MWCO = 3.5 and 14 kDa) were purchased from Spectrum Europe (Breda, Netherlands). ACVA was purified by recrystallization from methanol. Hydroquinone monomethyl ether (inhibitor) was removed from *N,N*-dimethylacrylamide (DMA) by purification on a basic alumina column. Ultrapure Q-water ultra-filtered on a Milli-Q Gradient A10 system (Millipore; Molsheim, France) was used in the experiments.

### Preparation of PDMA copolymers by reversible addition-fragmentation chain-transfer (RAFT) polymerization

Statistical poly(*N,N*-dimethylacrylamide-*co*-*tert*-butyl[2-(acryloylamino)ethyl]carbamate) [P(DMA-AEC-Boc)] or poly(*N,N*-dimethylacrylamide-*co*-2-(acryloylamino)-2-methylpropane-1-sulfonic acid) [P(DMA-AMPS)] copolymers with 95/5 molar ratio were prepared by RAFT copolymerization of DMA (12.1 mmol) and AEC-Boc (0.067 mmol) or AMPS (0.067 mmol) in ethanol (0.423 g of monomers per ml of solvent), respectively. The reaction was initiated with ACVA (0.035 mmol) and DMP (0.155 mmol) was used as CTA. The mixtures were purged with argon for 20 min and polymerized at 70 °C for 30 min. Resulting P(DMA-AEC-Boc) and P(DMA-AMPS) polymers were purified by repeated precipitation in tenfold excess of hexane. The composition of reactive copolymers was determined by ^1^H NMR spectroscopy (see Supporting Information SI; Figs. S1 and S2).

### Preparation of fluorescently labeled P(DMA-AEC-Boc) and P(DMA-AMPS)

Methanolic solutions (2 ml) of P(DMA-AEC-Boc) or P(DMA-AMPS) (0.3 g) and sodium borohydride (20 mg) were stirred at room temperature (RT) for 2 h under an argon atmosphere to remove RAFT leaving group. Resulting intermediates were purified on a Sephadex LH-20 chromatographic column with methanol as an eluent under argon purging; the solvent was removed using a vacuum rotary evaporator. Afterwards, methanolic solutions of copolymers (150 mg; 33 mg/ml) and DY-615-maleimide (0.1 mg; 0.1 mg/ml) were added and the mixture was stirred at RT for 16 h. The fluorescently labeled P(DMA-AEC-Boc)-DY-615 or P(DMA-AMPS)-DY-615 polymers were purified by gel filtration on a Sephadex LH-20 column with methanol as an eluent; the solvent was then vacuum-evaporated at RT. Amount of labeled DY-615 was determined by UV–Vis spectrophotometry at 621 nm (molar absorption coefficient *ε* = 200,000 l/mol·cm).

### Preparation of NHS-activated and alendronate-modified P(DMA-AEC-Boc)-DY-615 and P(DMA-AMPS)-DY-615 copolymers

NHS-activated P(DMA-AEC-Boc)-DY-615 and P(DMA-AMPS)-DY-615 copolymers were synthesized from P(DMA-AEC-Boc)-DY-615 and P(DMA-AMPS)-DY-615, respectively, using Steglich esterification via activation of carboxyl-end groups with DCC/NHS^36^. The synthesis was followed by the reaction with amino groups of Ale. The NHS-activated P(DMA-AEC-Boc)-DY-615 was obtained from P(DMA-AEC-Boc)-DY-615 (0.018 mmol) added to acetone solution (8 ml) of NHS (0.09 mmol), DMAP catalyst (2.5 µmol), and DCC (0.09 mmol; fivefold excess to polymer) at 5 °C. NHS-activated P(DMA-AMPS)-DY-615 was prepared with a higher excess of NHS, DCC, and DMAP compared to that of NHS-activated P(DMA-AEC-Boc)-DY-615. Briefly, cold acetone solution (5 ml; 5 °C) of NHS (0.18 mmol), DMAP catalyst (1.1 µmol), and DCC (0.18 mmol; 20-fold excess to the polymer) was added to P(DMA-AMPS)-Dy-615 (9.1 µmol) and the reaction continued at RT for 12 h with stirring under argon atmosphere. Precipitated byproduct (dicyclohexylurea) was filtered-off via a Millex-HA syringe filter (0.45 μm pore size) and acetone was evaporated under vacuum. NHS-activated P(DMA-AEC-Boc)-DY-615 or P(DMA-AMPS)-DY-615 (0.018 mmol) was added to PB solution (5 ml; pH 7.4) of alendronate sodium trihydrate (Ale; 0.18 mmol) at 5 °C; the mixture was then vigorously stirred at RT for 48 h and dialyzed against water (MWCO 3.5 kDa) for 48 h and freeze-dried. The presence of phosphonate groups in the resulting copolymers was confirmed by ^31^P NMR spectroscopy: δ 18.1 ppm for Ale-P(DMA-AEC-Boc)-DY-615 or 18.1 and 17.8 ppm for Ale-P(DMA-AMPS)-DY-615 (SI, Fig. S3).

### *N*-Boc deprotection of Ale-P(DMA-AEC-Boc)-DY-615

Ale-P(DMA-AEC-Boc)-DY-615 (1 mmol) was dissolved in 3 M hydrochloric acid in methanol (3 ml) and the mixture was stirred at RT for 2 h. Methanol was removed on a rotary evaporator and resulting Ale-P(DMA-AEC)-DY-615 was dialyzed against water (MWCO 3.5 kDa) for 48 h and lyophilized.

### Synthesis of NaYF_4_:Yb,Er core nanoparticles (C-UCNP)

C-UCNP were synthesized according to previously published procedures^37,38^. Briefly, yttrium(III), ytterbium(III), and erbium(III) chlorides (1 mmol; 0.78/0.2/0.02 mol/mol/mol, respectively) and oleic acid (6 ml) were dissolved in octadec-1-ene (15 ml) at 160 °C for 30 min under an argon atmosphere. The mixture was cooled down to RT to allow addition of methanolic NaOH solution (2.5 mmol) and NH_4_F (4 mmol). The temperature was then increased to 70 °C to evaporate methanol and subsequently to 300 °C for 1.5 h to produce C-UCNP. They were separated by centrifugation (3,460 rcf) for 30 min, washed in hexane/ethanol mixture (1:1 v/v) twice (14 ml each), and dispersed in hexane.

### Synthesis of NaYF_4_:Yb,Er@NaYF_4_:Nd core–shell nanoparticles (CS-UCNP)

Analogously to the above procedure, CS-UCNP were prepared using yttrium(III) (0.4 mmol) and neodymium(III) chloride (0.1 mmol) and oleic acid (6 ml) dissolved in octadec-1-ene (15 ml). The mixture was heated at 160 °C for 30 min under an argon atmosphere, cooled down to RT, and hexane dispersion (15 ml) of NaYF_4_:Yb,Er nanoparticles (150 mg) and methanolic solution of NaOH (1.25 mmol) and NH_4_F (2 mmol) were added. Methanol and hexane were evaporated at 70 °C and the mixture was heated at 300 °C for 1.5 h under an argon atmosphere. The nanoparticles were separated by centrifugation (3,460 rcf) for 30 min and washed in hexane/ethanol, ethanol, ethanol/water, and finally water.

### Synthesis of CS-UCNP@Ner-PEG

Surface of the CS-UCNP was modified by Ner-PEG according to an earlier published report^39^. Ner-PEG (3.5 mg) was added to an aqueous dispersion of CS-UCNP (6 ml; 1.7 mg/ml) and the mixture was stirred at RT for 12 h. Resulting CS-UCNP@Ner-PEG were dialyzed against water using a cellulose membrane (MWCO 14 kDa) to remove excessive PEG-Ner.

### Modification of CS-UCNP with Ale-PDMA-DY-615, Ale-P(DMA-AMPS)-DY-615, and Ale-P(DMA-AEC)-DY-615

Dispersion of CS-UCNP (5 ml; 1.1 mg/ml of water) was mixed with Ale-PDMA-DY-615, Ale-P(DMA-AMPS)-DY-615, or Ale-P(DMA-AEC)-DY-615 polymers (2.75 mg) with sonication (UP200S Hielscher Ultrasound Technology; Teltow, Germany) at 10 W for 5 min and stirring at RT for 12 h. The resulting PDMA-modified CS-UCNP, CS-UCNP@Ale-PDMA-DY-615, CS-UCNP@Ale-P(DMA-AMPS)-DY-615, and CS-UCNP@Ale-P(DMA-AEC)-DY-615 were dialyzed against water for 24 h using cellulose membrane (MWCO 3.5 kDa).

### Characterization of nanoparticles

The morphology of nanoparticles was analyzed using a Tecnai Spirit G2 transmission electron microscope (TEM; FEI; Brno, Czech Republic)^38^. The particle size and distribution were determined by measuring at least 300 nanoparticles from four random TEM micrographs using ImageJ software. The average diameter of ellipsoidal nanoparticles was determined as follows: long axis (morphological descriptor *MaxFeret*) and short axis (morphology descriptor *MinFeret*) were measured and the average diameter was approximated as *D* = 1/2*(*MaxFeret* + *MinFeret*). Number-(*D*_n_), weight-average diameter (*D*_w_), and the uniformity (dispersity *Ð*) were calculated as follows:

1 $$D_{{\text{n}}} = \sum {\text{N}}_{{\text{i}}} D_{{\text{i}}} /\sum {\text{N}}_{{\text{i}}}$$

2 $$D_{{\text{w}}} = \sum {\text{N}}_{{\text{i}}} D_{{\text{i}}}^{{4}} /\sum {\text{N}}_{{\text{i}}} D_{{\text{i}}}^{{3}}$$

3where N_i_ and *D*_i_ are number and diameter of the nanoparticle, respectively.

The X-ray powder diffraction (XRD) measurements were performed using an Explorer powder diffractometer (GNR Agrate Conturbia, Italy) in the region 13–80 degree 2*Θ*.

The hydrodynamic nanoparticle diameter (*D*_h_), size distribution (polydispersity *PD*), and ζ-potential were determined by dynamic light scattering (DLS) on a Zetasizer Ultra Instrument (Malvern Instruments; Malvern, UK) at 25 °C; *D*_h_ and *PD* were calculated from the intensity-weighted distribution function obtained by CONTIN analysis of the correlation function embedded in Malvern software.

^1^H and ^31^P NMR spectra were recorded using a Bruker Avance III 600 spectrometer (Bruker; Billerica, MA, USA) equipped with a 5 mm diffusion probe-head. ^1^H NMR conditions were as follows: 90° pulse width 10 μs, acquisition time 4.54 s, spectral width 7,212 Hz, relaxation delay 10 s, and 32 scans. ^31^P NMR spectra were recorded in D_2_O at 22 °C with 90° pulse, width 18 μs, relaxation delay 15 s, spectral width 36,232 Hz, and acquisition time 0.9 s. The resulting spectra were processed in Topspin 4.1.0 software, where the integrated intensities were determined with an accuracy of ± 1%. During the measurements, temperature was maintained within ± 0.2 K using a BVT 3000 temperature unit.

Weight-(*M*_w_), number-average molar mass (*M*_n_), and *M*_w_/*M*_n_ of the polymers were determined by the size exclusion chromatography (SEC) on a Shimadzu HPLC system (Tokyo, Japan) equipped with a UV–Vis diode array and OptilabrEX refractive index and DAWN EOS multiangle light scattering detectors (Wyatt; Santa Barbara, CA, USA). A TSK SuperAW3000 column was used with methanol/sodium acetate buffer (80/20 v/v) as a mobile phase (pH 6.5) at flow rate of 0.6 ml/min. FTIR spectra were recorded on a 100 T FTIR spectrometer (Perkin-Elmer; Waltham, MA, USA) using a Specac MKII Golden Gate single attenuated total reflection (ATR). The content of DY-615 in methanolic solution of polymers was determined using a Specord Plus UV–Vis spectrometer (Analytik Jena, Germany) at 621 nm using the molar absorption coefficient for DY-615 at 621 nm (*ε* = 200,000 l/mol cm). The elemental composition of particles was obtained from energy-dispersive X-ray (EDX) analysis (EDAX detector; Mahwah, NJ, USA).

The upconversion luminescence spectra of C-UCNP and CS-UCNP and their PDMA- or PEG-coated analogues (1 mg/ml) were measured in a Hellma 114F-QS cuvette (10 × 4 mm path length; Sigma-Aldrich) at RT using a FS5 spectrofluorometer (Edinburgh Instruments; Livingston, UK) equipped with continuous xenon lamps (150 W) and CW 808 and 980 nm infrared diode lasers as an excitation source with nominal laser power of 2 W (MDL-III-808 and MDL-III-980; beam size of 5 × 8 mm^2^).

### In vitro distribution of nanoparticles in cells

Human hepatocellular carcinoma HepG2 (ECACC 85011430) and human cervix epitheloid carcinoma HeLa cells (ECACC 93021013) were cultivated in DMEM with 3 mM glutamine, 10% (v/v) fetal calf serum (Biosera; Nuaille, France), 10 mM HEPES, 100 IU/ml penicillin, 100 μg/ml streptomycin, and 5 mM glucose at 37 °C in humidified air with 5% CO_2_. Rat insulinoma INS-1E cells (kind gift from Prof. Maechler, the University of Geneva) were cultured in 11 mM glucose and RPMI 1640 medium supplemented with 5% (v/v) fetal calf serum, 10 mM HEPES, 1 mM pyruvate, 50 μM mercaptoethanol, 50 IU/ml penicillin, and 50 μg/ml streptomycin. The cells were then cultured on poly(L-lysine)-coated glass coverslips in DMEM (2 ml) for 2 days, incubated with the nanoparticles (150 μl; 0.43–0.93 mg/ml) for 24 h, and transferred in a thermostable chamber at 37 °C under 5% CO_2_ atmosphere, mimicking normal cultivation conditions. Finally, the HeLa, HepG2, and INS-1E cells were observed in a Leica TCS SP8 AOBS confocal inverted fluorescent microscope (Wetzlar, Germany) equipped with an objective HC PL APO 63 ×/1.20 NA W CORR CS2, WD = 0.3 mm. The particles were excited by a Chameleon Ultra I pulsed infrared tunable laser with wavelength range 690–1040 nm, maximum output power 4 W, pulse frequency 80 MHz, pulse width ~ 140 fs, and laser intensity controlled by an electrooptical EOM modulator (Coherent; Santa Clara, CA, USA) and attenuator at 808 and 980 nm excitation.

### Cytotoxicity of nanoparticles

The cytotoxicity of particles was measured using a trypan blue exclusion test (Thermo Fisher Scientific). Briefly, HeLa, HepG2, and INS-1E cells were cultured in a cell medium at 37 °C for 48 h under 5% CO_2_ humidified atmosphere and incubated with the particles (0.01, 0.02, 0.05, 0.1, and 0.2 mg/ml) for 24 h under the same atmosphere. In vitro cell viability was determined by 0.4% trypan blue staining and the fraction of living cells was counted on a Countess automated cell counter (Thermo Fisher Scientific).

## Results and discussion

### Reversible addition-fragmentation chain-transfer (RAFT) polymerization of DMA and its copolymerization with AEC-Boc and AMPS

**Figure 1:**
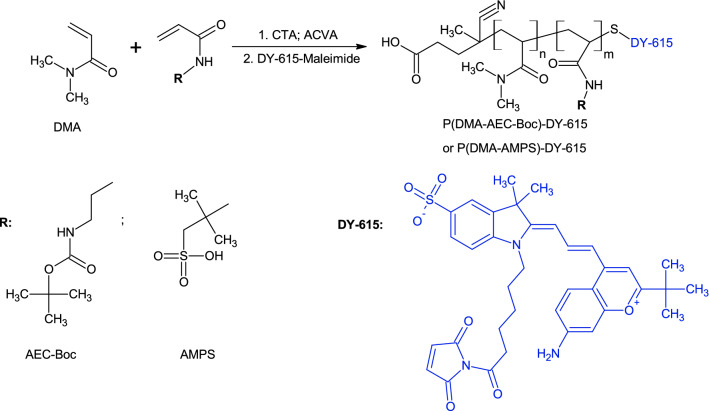
Copolymerization of *N,N*-dimethylacrylamide (DMA) with *tert*-butyl[2-(acryloylamino)ethyl]carbamate (AEC-Boc) or 2-(acryloylamino)-2-methylpropane-1-sulfonic acid (AMPS) and labeling with DY-615 fluorescent dye resulting in P(DMA-AMPS)-DY-615 or P(DMA-AEC-Boc)-DY-615 copolymers. CTA—chain transfer agent, ACVA—4,4′-azobis(4-cyanovaleric acid).

As the starting UCNP are generally hydrophobic due to stabilization by OA, their surface hydrophilization is required. Here, poly(*N,N*-dimethylacrylamide) (PDMA) was selected as a basic coating polymer of the particles due to its excellent solubility in water, as well as in organic solvents, biocompatibility, and last but not least good reactivity^40^. To obtain PDMA with a controlled and narrow distribution of molar mass (*M*_w_/*M*_n_ < 1.2), which is important for design of nanocarriers possessing uniform physicochemical properties and reproducible biological experiments, RAFT polymerization was used. The technique enables easy removal of thiocarbonyl end-group and subsequent conjugation of a therapeutic agent^41,42^. Therefore, DMA was copolymerized by RAFT polymerization with two reactive comonomers, AEC-Boc or AMPS (95/5 mol/mol) carrying amino and sulfo groups with positive and negative charges, respectively (Fig. 1). The charge is one of the important parameters that affect cellular internalization of the particles.

Polymerizations were terminated at 85–86% conversions (according to ^1^H NMR), yielding *M*_n_ ~ 11 kDa and a narrow molar mass distribution for both P(DMA-AEC-Boc) and P(DMA-AMPS). These values agreed with calculated *M*_n,th_ (Table 1) and were sufficiently high to ensure a good steric stabilization of the particles in aqueous media.

**Table 1:** Characterization of polymers used for modification of UCNP.

Polymer	Conversion^a^ (%)	Reactive comonomer^a^ (mol%)	*M*_n,th_^b^ (kDa)	*M*_n_^b^ (kDa)	*M*_w_^b^ (kDa)	*M*_w_/*M*_n_^b^	DY-615^c^ (wt%)
PDMA	98	–	10.5	8.7	10.95	1.27	1.51
P(DMA-AEC-Boc)	85	5.3	11.26	11.0	11.61	1.06	0.48
P(DMA-AMPS)	86	5.0	11.47	11.4	12.03	1.06	0.52

According to ^a1^H NMR, ^b^size exclusion chromatography, ^c^UV–Vis spectroscopy in methanol (Ɛ = 200,000 l/mol·cm); *M*_n,th_—theoretical molar mass, *M*_n_ and *M*_w_—number- and weight-average molar mass, respectively; PDMA—poly(*N,N*-dimethylacrylamide), AEC-Boc—*tert*-butyl[2-(acryloylamino)ethyl]carbamate, AMPS—2-(acryloylamino)-2-methylpropane-1-sulfonic acid.

**Figure 2:**
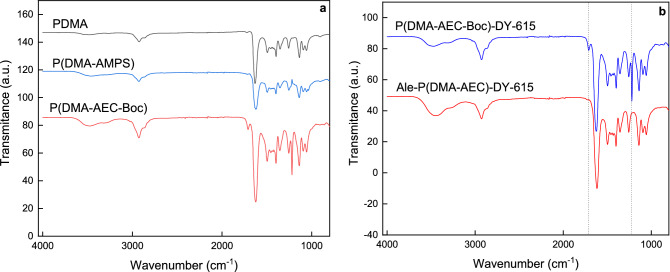
ATR FTIR spectra of (**a**) PDMA, P(DMA-AMPS), P(DMA-AEC-Boc) and (**b**) Ale-P(DMA-AEC)-DY-615 after deprotection of amino groups.

Let us note that poor colloidal stability and aggregation of PDMA-coated particles was observed for *M*_w_ < 8 kDa, whereas higher molar mass provided effective stabilization^36^. Moreover, the obtained molar masses of both PDMA-based polymers were lower than the renal excretion limit that is generally considered to be < 40 kDa^43^. Copolymer compositions were analyzed by ^1^H NMR spectroscopy from integrated intensities of AEC-Boc (CH_3_)_3_ methyl protons (signal ‘d’) and DMA (CH_3_)_2_ methyl protons (signal ‘c’) (Fig. S1 a,b). Content of AMPS in the copolymer was calculated from signal ‘d’ (Fig. S2 a,b). Amount of reactive monomers in P(DMA-AEC-Boc) and P(DMA-AMPS) was 5.3 and 5.0 mol.%, respectively, which was in agreement with the monomer feed ratio (95/5 mol/mol). In the FTIR spectrum of P(DMA-AEC-Boc), peaks observed at ~ 1710 and 1220 cm^−1^ were assigned to ν(C=O) and ν(C–O) stretching vibrations of Boc group, respectively (Fig. 2a). The corresponding peaks of AMPS unit in P(DMA-AMPS) at 1205, 1180, and 1036 cm^−1^ were attributed to ν(S=O) stretching vibrations. Both ^1^H NMR and FTIR spectra thus confirmed successful preparation of P(DMA-AEC-Boc) and P(DMA-AMPS) copolymers.

### Functionalization of PDMA (co)polymer with DY-615 dye and alendronate

**Figure 3:**
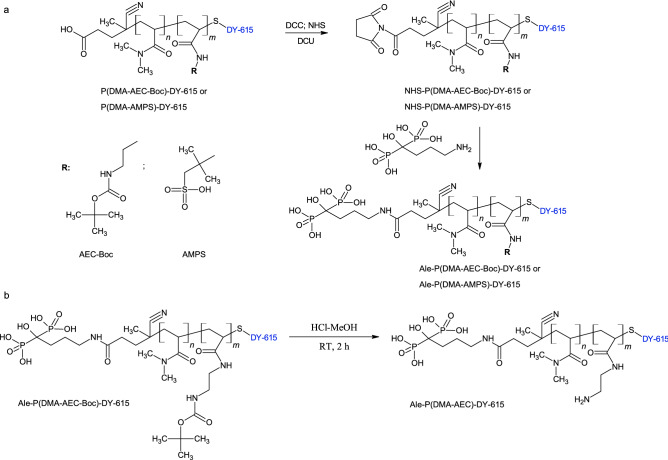
(**a**) Activation of carboxyl-end groups of P(DMA-AEC-Boc)-DY-615 or P(DMA-AMPS)-DY-615 with *N*-hydroxysuccinimide (NHS) and *N*,*N*′-dicyclohexylcarbodiimide (DCC) and reaction with alendronate (Ale). (**b**) *N*-Boc deprotection of Ale-P(DMA-AEC-Boc)-DY-615. DCU—dicyclohexylurea.

In order to monitor the fixation of polymer to the nanoparticle surface, PDMA (co)polymer was modified with DY-615 stain. In the first step, P(DMA-AEC-Boc) and P(DMA-AMPS) containing SH end-groups were labeled with DY-615 fluorescent dye (Fig. 1). According to UV–Vis spectroscopy, the content of DY-615 in PDMA-DY-615, P(DMA-AEC-Boc)-DY-615 and P(DMA-AMPS)-DY-615 was 1.51, 0.48 and 0.52 wt%, respectively, with the conversions 96 wt% for PDMA-DY-615 and ranging 47–53 wt% for P(DMA-AEC-Boc)-DY-615 and P(DMA-AMPS)-DY-615. According to literature, such amount of dye was sufficient for fluorescent labeling^44^. In the next step, the PDMA (co)polymer was functionalized with Ale to ensure steric stabilization of the NaYF_4_:Yb,Er@NaYF_4_:Nd nanoparticles in biological media (Fig. 3a). It is an advantage that Ale contains bisphosphonate moieties with a strong binding affinity to a number of metal ions, such as alkaline earth^45,46^ and transition metals^47^, as well as lanthanides^48^. The presence of phosphonate groups in the Ale-P(DMA-AEC-Boc)-DY-615 and Ale-P(DMA-AMPS)-DY-615 was confirmed by ^31^P NMR spectroscopy (Fig. S3). In order to obtain positively charged particles, Boc-protected amino groups of Ale-P(DMA-AEC-Boc)-DY-615 polymer were removed (Fig. 3b) as confirmed by ^1^H NMR (Fig. S1c) and FTIR spectroscopy due to disappearance of peaks at 1710 and 1220 cm^−1^ assigned to Boc groups (Fig. 2b).

### Ale-P(DMA-AEC)-DY-615-, Ale-P(DMA-AMPS)-DY-615-, Ale-PDMA-, and PEG-Ner-modified CS-UCNP

**Figure 4:**
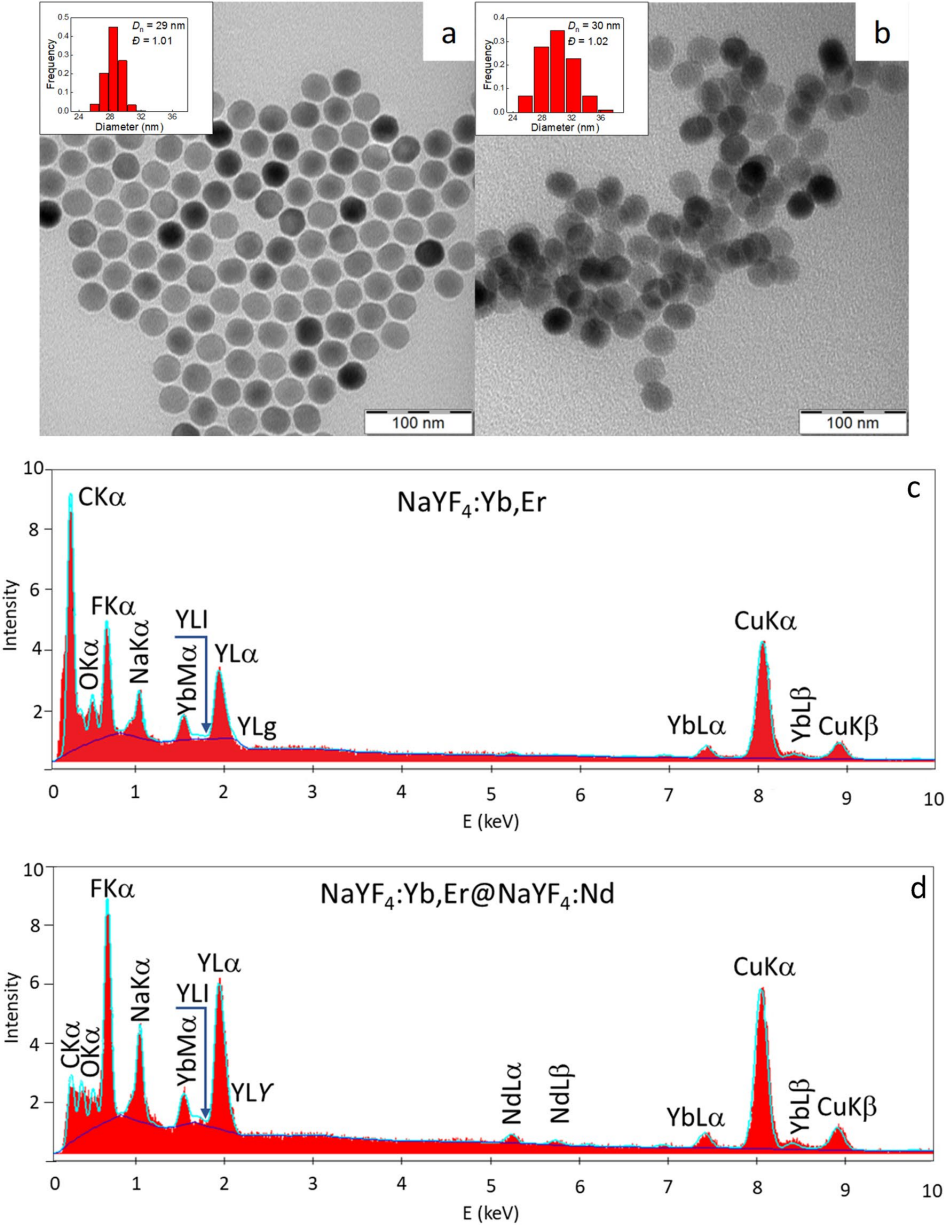
TEM micrographs and TEM/EDX analysis of (**a**, **c**) C-UCNP and (**b**, **d**) CS-UCNP. C-UCNP—NaYF_4_:Yb,Er; CS-UCNP—NaYF_4_:Yb,Er@NaYF_4_:Nd.

The uniformly-sized C-UCNP were prepared by a high-temperature (300 °C) coprecipitation of lanthanide chlorides in octadec-1-ene solvent in the presence of oleic acid as a stabilizer. According to TEM, the particles were spherical in shape with *D*_n_ = 29 nm and a narrow size distribution (*Ð* = 1.01; Fig. 4a). Such a narrow distribution is important in terms of the same physicochemical and biological properties and reproducibility of the results. Further, C-UCNP were covered with NaYF_4_:Nd shell containing an additional sensitizer (Nd^3+^) to enable excitation at 808 nm within the transparent NIR optical window of biological tissues and provide a bright NIR emission. The OA-stabilized CS-UCNP were ellipsoidal (Fig. 4b), in agreement with earlier described results^39^.

Both C-UCNP and CS-UCNP were characterized also by XRD (Fig. S4). Despite the fact that the intensities of CS-UCNP diffractograms were higher than those of C-UCNP, they were similar, corresponding to the standard β-NaYF_4_ known from literature (JCPDS card. No 28–1192). The full width at half maximum (FWHM) of several peaks for both samples was roughly the same and the crystal size from the first peak was estimated to 22 nm using the Scherrer formula:

$$D = \frac{K\,\lambda }{{FWHM\,\cos (\theta )}}$$

Here, *K* is the shape factor ~ 0.92 rad, *Θ* is the diffraction angle, and λ is the X-ray wavelength (0.154 nm). The only significant difference was the peak at 31.74 deg and three possible negligible peaks.

**Figure 5:**
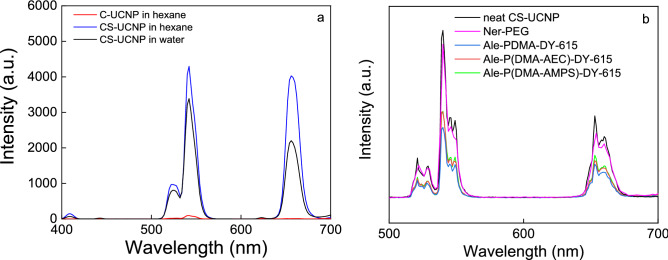
Photoluminescence upconversion spectra of neat C-UCNP and CS-UCNP in (**a**) hexane and water and (**b**) PEG-Ner-, Ale-P(DMA-AEC)-DY-615-, Ale-P(DMA-AMPS)-DY-615-, and Ale-PDMA-Dy-615-coated CS-UCNP particles in water at 808 nm excitation; particle concentration 1 mg/ml; power density 1 W/cm^2^. C-UCNP—NaYF_4_:Yb,Er; CS-UCNP—NaYF_4_:Yb,Er@NaYF_4_:Nd.

The upconversion luminescence of C-UCNP and CS-UCNP was determined by emission at 980 and 808 nm (Figs. 5 and S5). The emission of both core and core–shell nanoparticles exhibited the characteristic emission at 409 (^2^H_9/2_ → ^4^I_15/2_), 525 (^2^H_11/2_ → ^2^I_15/2_), 542 (^4^S_3/2_ → ^2^I_15/2_) and 656 nm (^4^F_9/2_ → ^2^I_15/2_) typical for the transitions of Er^3+^ ions in upconverting nanomaterials (Fig. 5a and S5). While the C-UCNP did not show upconversion emission under the 808 nm excitation, the incorporation of Nd^3+^ into the shell provided luminescence and excitation deep in the tissue.

As expected, compared to the C-UCNP, introduction of the NaYF_4_:Nd shell fourteen times increased emission intensity at 980 nm excitation with low power density (1 W/cm^2^; Fig. S5a). This demonstrated that the shell protected dopants in the core from quenching. The transfer of nanoparticles from hexane into water slightly decreased the emission intensity of CS-UCNP (Figs. 5a and S5b). The TEM/EDX spectrum of C-UCNP exhibited main peaks of Na, Y, and F elements and weaker Yb peak and C and Cu peaks from the standard supporting TEM grid (Fig. 4c). The spectrum of CS-UCNP differed by the appearance of small peaks at 5.3 and 5.8 keV, which proved the presence of neodymium in the shell layer^38^ (Fig. 4d). The next modification of CS-UCNP consisted of two steps: (*i*) carful removal of residual organic compounds (OA and octadec-1-ene) from particles by their washing with hexane, ethanol, and water and (*ii*) coating of particles with PDMA- or PEG-based (co)polymers. Coating of similar lanthanide-based nanoparticles by poly(*N,N*-dimethylacrylamide) copolymer was confirmed by FTIR analysis in our previous paper^49^. The resulting surface-modified particles varied in ζ-potential (Table 2), as the P(DMA-AMPS) and P(DMA-AEC) polymers contained sulfo and amino groups, respectively, rendering negative (− 9 mV) or positive ζ-potential (24 mV). In contrast, Ner-PEG and Ale-PDMA-DY-615 provided moderately positive surface charge (15 and 12 mV, respectively) to the particles. *D*_h_ of neat CS-UCNP (230 nm) reflected rather the size of aggregates than that of individual nanoparticles.

**Table 2:** Characterization of polymer-coated CS-UCNP.

Coating	*D*_h_ (nm)	*PD*	ζ-potential (mV)
Ale-PDMA-DY-615	113	0.20	12
Ale-P(DMA-AEC)-DY-615	175	0.11	24
Ale-P(DMA-AMPS)-DY-615	164	0.13	-9
PEG-Ner	168	0.16	15
–	230	0.15	30

*D*_h_ - hydrodynamic diameter in water, *PD* - polydispersity index (DLS); CS-UCNP—NaYF_4_:Yb,Er@NaYF_4_:Nd.

After the modification with Ale-PDMA-DY-615, Ale-P(DMA-AEC)-DY-615, and Ale-P(DMA-AMPS)-DY-615, *D*_h_ of particles decreased to 113, 175, and 164 nm, respectively (Table 2). In contrast, the size of Ner-PEG-coated nanoparticles decreased from 230 to 168 nm (Table 2), which was in accordance with earlier published results on the PEGylated UCNP with extraordinarily good colloid stability in PBS^34^. The polydispersity of particles measured by DLS was moderate (*PD* = 0.11–0.20; Table 2). After the modification of CS-UCNP with Ner-PEG-, Ale-PDMA-DY-615, and Ale-P(DMA-AEC)-DY-615, ζ-potential of pure CS-UCNP containing bare lanthanide atoms decreased from the highly positive charge (30 mV) to 15, 12, and 24 mV, respectively. In contrast, the negative surface charge (− 9 mV) on Ale-P(DMA-AMPS)-DY-615-modified particles was likely caused by the presence of sulfo groups. The presence of DY-615 dye on Ale-P(DMA-AEC)-DY-615, Ale-PDMA-DY-615, and Ale-P(DMA-AMPS)-DY-615-coated CS-UCNPs was proved by the appearance of absorption peak at 617 nm in the UV–Vis spectra (Fig. S6a). The functionalization of particles with Ner-PEG and PDMA-based (co)polymers was also confirmed by the photoluminescence spectra of CS-UCNP (Figs. 5b and S6b). While the free DY-615 emitted light at 641 nm, after the excitation of Ale-PDMA-DY-615-, Ale-P(DMA-AEC)-DY-615-, and Ale-P(DMA-AMPS)-DY-615-coated CS-UCNP at 621 nm, the intense emission at 637 nm was assigned to DY-615 (Fig. S6b). The shift of emission peak (by 3 nm) from that of free DY-615 was ascribed to the conjugation of DY-615 with polymer. The modification of CS-UCNP with polymers slightly decreased the upconverting emission intensity at 808 nm excitation (Fig. 5b).

### Labeling of carcinoma cells with neat, Ale-P(DMA-AEC)-DY-615-, Ale-P(DMA-AMPS)-DY-615-, PDMA-Ale-, and PEG-Ner-modified CS-UCNP

The internalization of both neat and Ale-P(DMA-AEC)-DY-615-, Ale-P(DMA-AMPS)-DY-615-, PDMA-Ale-, and PEG-Ner-modified CS-UCNP was investigated on the human cervix epitheloid carcinoma HeLa, human hepatocellular carcinoma HepG2, and rat insulinoma INS-1E cells. While the HeLa cells are commonly investigated in biomedical cancer research including experimental PDT, the HepG2 immortal cell line derived from the liver tissue represents a model system of liver metabolism and for drug targeting. Moreover, the INS-E1 cell line originally established from rat insulinoma is a useful model of pancreatic islet β-cell function associated with diabetes.

**Figure 6:**
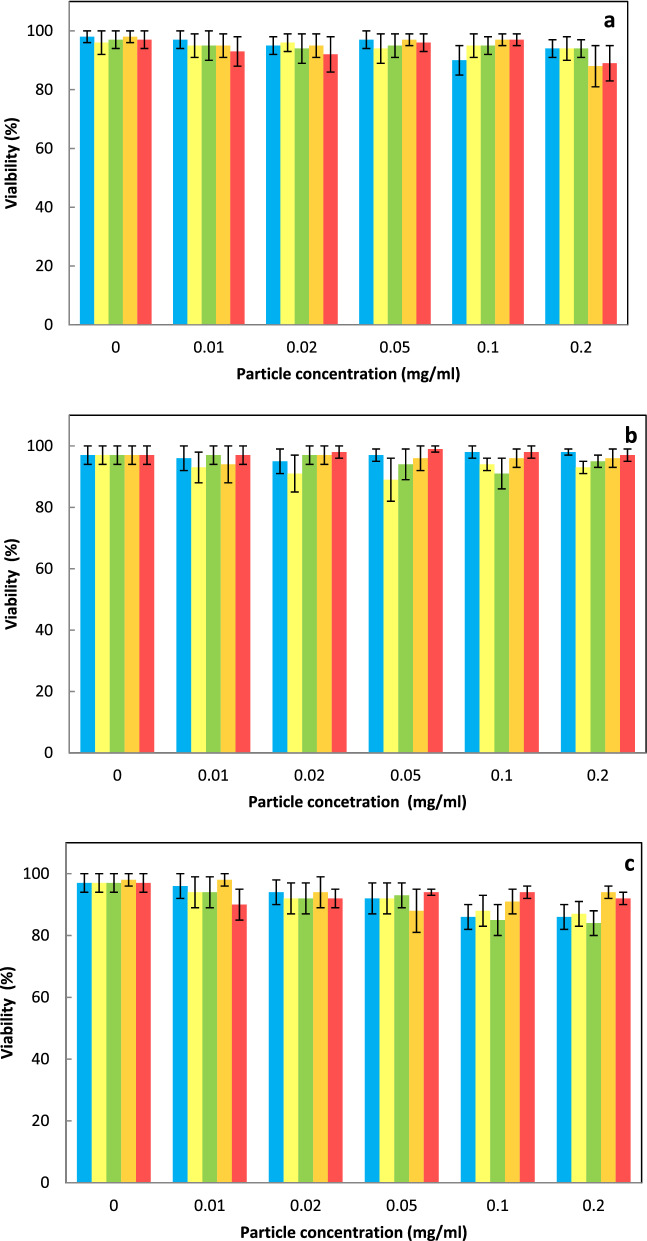
Viability of (**a**) HeLa, (**b**) HepG2, and (**c**) INS-1E cells incubated with CS-UCNP (blue), CS-UCNP@Ner-PEG (yellow), CS-UCNP@Ale-P(DMA-AEC)-DY-615 (green), CS-UCNP@Ale-PDMA-DY-615 (orange), and CS-UCNP@Ale-P(DMA-AMPS)-DY-615 (red) for 24 h.

At the beginning, the cytotoxicity of nanoparticles (0.01, 0.02, 0.05, 0.1, and 0.2 mg/ml) was determined after their incubation with HeLa, HepG2, and INS-1E cells for 24 h (Fig. 6).

**Figure 7:**
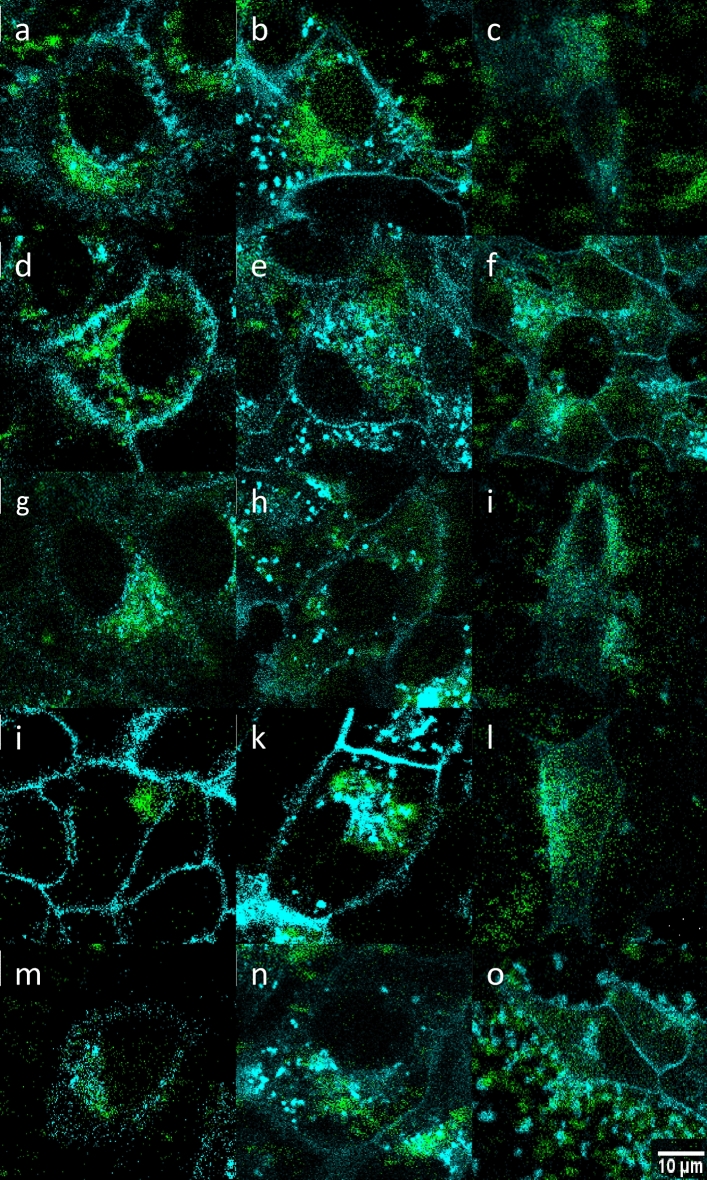
Detailed confocal micrographs showing distribution of (**a**–**c**) CS-UCNP, (**d**–**f**) CS-UCNP@Ner-PEG, (**g**–i) CS-UCNP@Ale-P(DMA-AEC)-DY-615, (**j**–**l**) CS-UCNP@Ale-PDMA-DY-615, and (**m**–**o**) CS-UCNP@Ale-P(DMA-AMPS)-DY-615 (1–1.7 mg/ml) in (**a**, **d**, **g**, **j**, **m**) HeLa, (**b**, **e**, **h**, **k**, **n**) Hep-G2, and (**c**, **f**, **i**, **l**, **o**) INS-1E cells after excitation at 980 nm (laser power 30–50 mW); cell membranes are blue and nanoparticles are green. CS-UCNP—NaYF_4_:Yb,Er@NaYF_4_:Nd.

The cell viability did not change after exposure to particles even at the concentration of 0.2 mg/ml that was higher than that used in other biological experiments. The emission spectra of Ale-P(DMA-AEC)-DY-615-, Ale-P(DMA-AMPS)-DY-615-, and PDMA-Ale-coated C-UCNP exhibited typical upconversion peaks at 530 and 650 nm (Fig. S7). After the engulfment of particles (0.15 mg/ml) by the cells, their components were also fluorescent. Nevertheless, due to the difference between the fluorescence of nanoparticles and cells, the particles were clearly detectable. It was obvious that the particles even in the cell milieu did not aggregate. Processing of selected segments from confocal micrographs was exemplified on the distribution of CS-UCNP@Ale-P(DMA-AEC)-DY-615 in the CellMask™-stained HepG2 cells (Fig. S8). When overviewing the segments of confocal micrographs of all particle-engulfed cells (Fig. 7), it was found that the core–shell nanoparticles with positive ζ-potential easily penetrated negatively charged cell membranes. The particles coated with PEG-Ner, Ale-P(DMA-AEC)-DY-615, and Ale-PDMA-DY-615 with the ζ-potential ranging 6–30 mV exhibited cellular uptake (Fig. 7a–l). In contrast, particles coated by Ale-P(DMA-AMPS)-DY-615 with negative surface charge (− 9 mV) seemed to be less prone to cell labeling (Fig. 7m–o).

**Figure 8:**
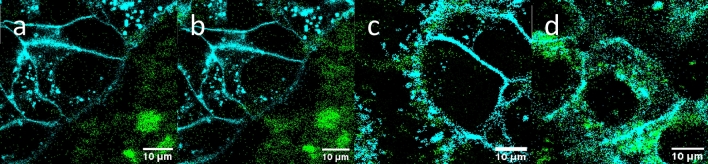
Gradual penetration of CS-UCNP@Ale-P(DMA-AEC)-DY-615 into HepG2 cells (**a**) 1, (**b**) 2, (**c**) 4, and (**d**) 24 h after incubation. CS-UCNP—NaYF_4_:Yb,Er@NaYF_4_:Nd.

During the experiments, the nanoparticles gradually penetrated the cells; after the first 2 h, they were mostly localized around the cells, not inside, but after 4 and 24 h, they adhered to the cell membranes entering then most cells, respectively. As an example, the penetration of CS-UCNP@Ale-P(DMA-AEC)-DY-615 into HepG2 cells was shown on the confocal micrographs (Fig. 8), confirming that the particles were noncytotoxic and biocompatible.

**Figure 9:**
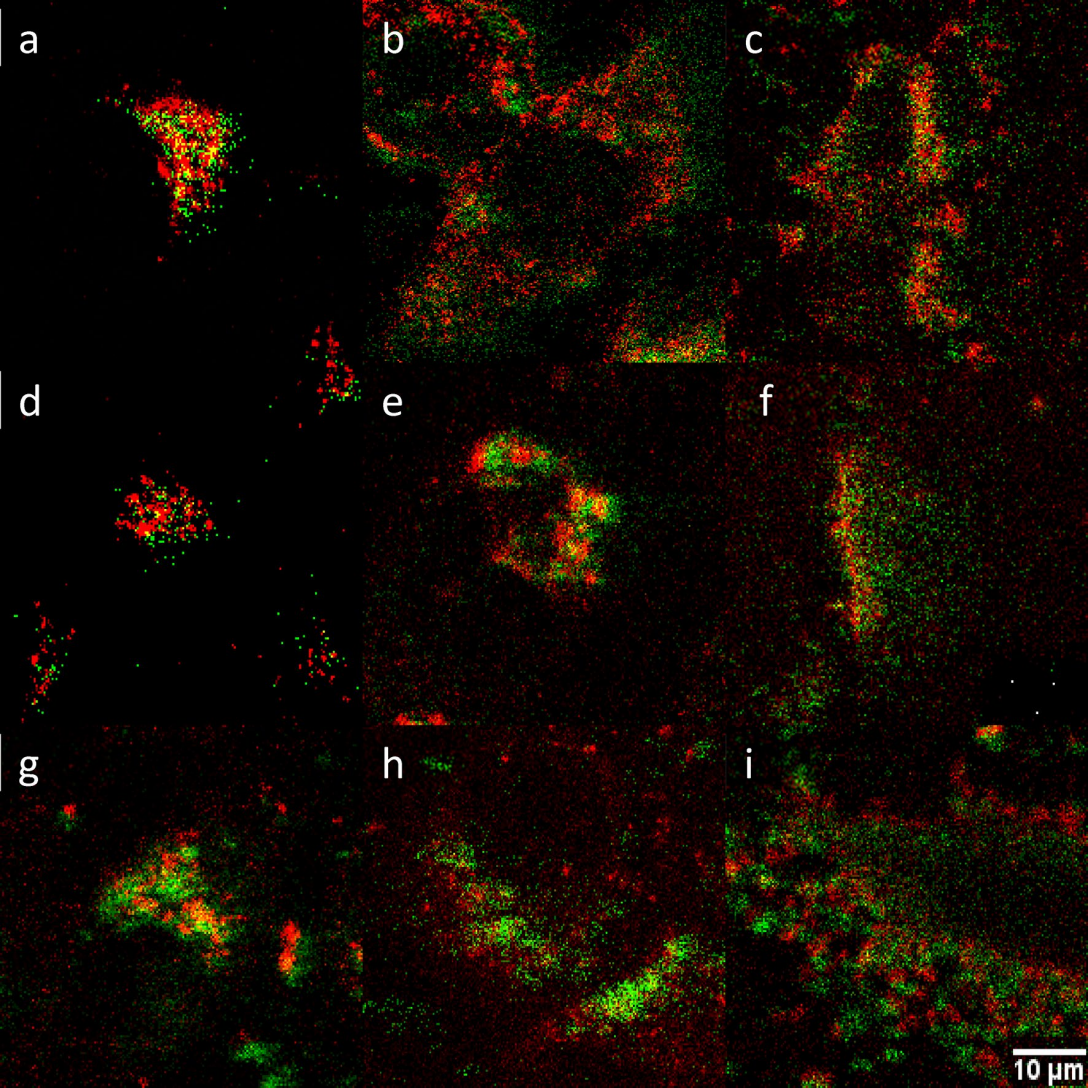
Overlay of luminescence of (**a**–**c**) CS-UCNP@Ale-P(DMA-AEC)-DY-615, (**d**–**f**) CS-UCNP@Ale-PDMA-DY-615, and (**g**–**i**) CS-UCNP@Ale-P(DMA-AMPS)-DY-615 with DY-615 in (**a**, **d**, **g**) HeLa, (**b**, **e**, **h**) HepG2, and (**c**, **f**, **i**) INS-1E cells. Excitation of the particles (green) and DY-615 (red) at 980 and 621 nm, respectively. CS-UCNP—NaYF_4_:Yb,Er@NaYF_4_:Nd.

In the cell cultures containing Ale-P(DMA-AMPS)-DY-615-, Ale-P(DMA-AEC)-DY-615-, and Ale-PDMA-DY-615-modified CS-UCNP in the absence of CellMask™ green, the particles and the polymer were green and red, respectively, proving that the coating remained firmly attached to the particle surface (Fig. 9). Some symmetrical shifts in the images can be ascribed to errors induced by switching between two different lasers.

**Figure 10:**
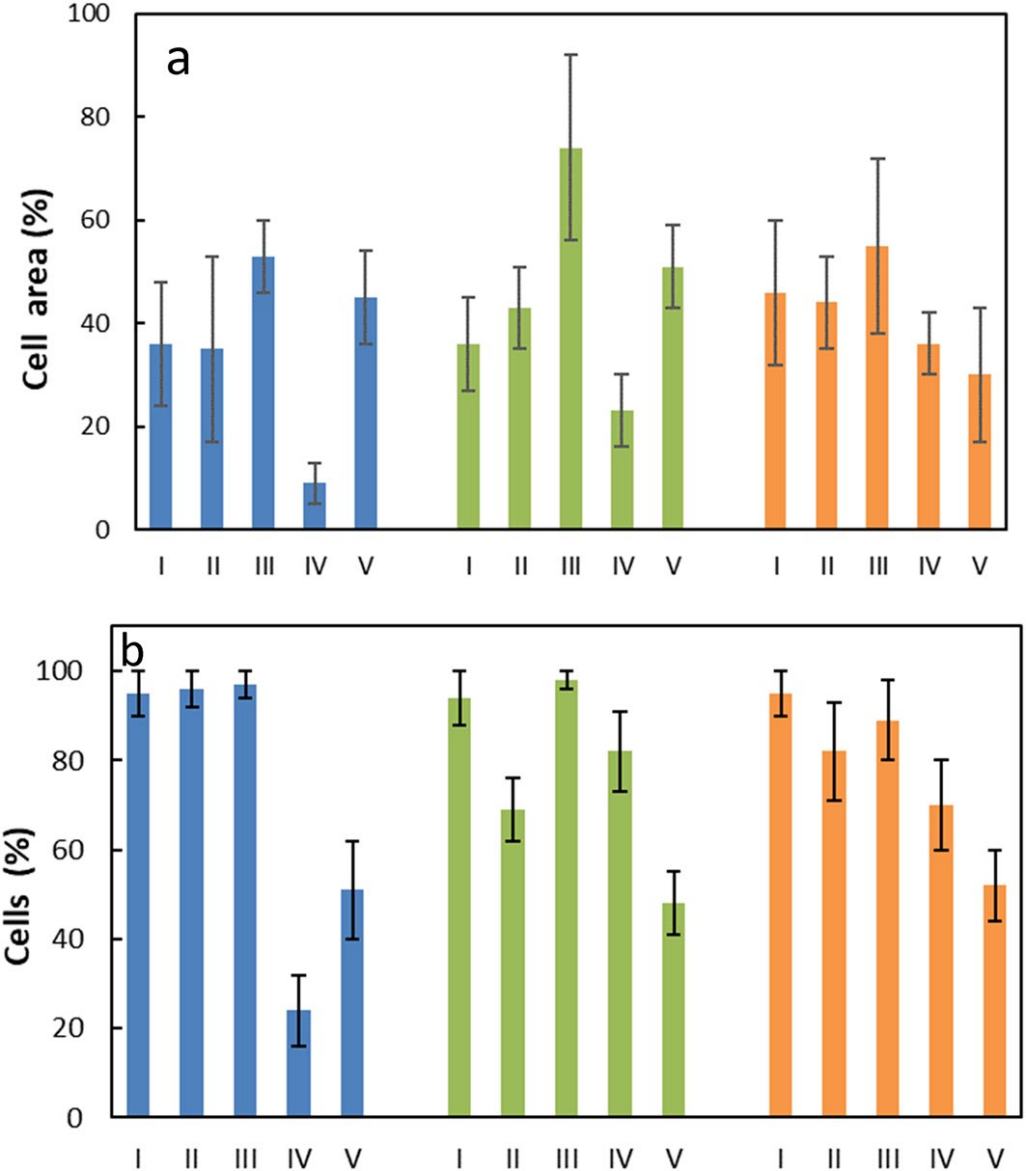
(**a**) HeLa (blue), HepG2 (green), and INS-1E cell area (orange) occupied by the (I) CS-UCNP, (II) CS-UCNP@Ner-PEG, (III) CS-UCNP@Ale-P(DMA-AEC)-DY-615, (IV) CS-UCNP@Ale-PDMA-DY-615, and (V) CS-UCNP@Ale-P(DMA-AMPS)-DY-615 and (**b**) percentage of cells containing the particles. CS-UCNP—NaYF_4_:Yb,Er@NaYF_4_:Nd.

To compare spreading of nanoparticles inside the cells and monitor the number of particle-internalized cells, the percentage of cell area occupied by nanoparticles and the percentage of cells containing the particles was determined (Fig. 10).

Considering both these parameters, CS-UCNP@Ale-P(DMA-AEC)-DY-615 were most spread inside the cells compared to other particle types and at the same time they penetrated all cells. In contrast, CS-UCNP@Ale-P(DMA-AMPS)-DY-615 with negative ξ-potential internalized only relatively small number of cells.

## Conclusions

Differently charged polymer-coated CS-UCNP, uniform in size with diameter of 29 nm, were synthesized by a high-temperature coprecipitation of lanthanide chlorides in a high-boiling organic solvent. This was followed by the modification of particles with four polymers: negatively charged sulfo group-containing P(DMA-AMPS), positively charged P(DMA-AEC), and two electroneutral PDMA and PEG as a control. Thanks to the PDMA-based coatings, the colloidal stability of particles in the cell culture medium was ensured. Optionally, the polymers were labeled with DY-615 and used as a coating of CS-UCNP to make fluorescent imaging of carcinoma cells possible, allowing at the same time to control the stability of both nanoparticles and coatings in the cell medium. All the particles, up to 0.2 mg/ml concentration, were very well tolerated by all three examined types of carcinoma cells, i.e., HeLa, HepG2, and INS-1E, without any sign of toxicity. The highest particle uptake in carcinoma cells was observed with CS-UCNP@Ale-P(DMA-AEC)-DY-615, followed by CS-UCNP@Ale-PDMA-DY-615, CS-UCNP@Ner-PEG, and neat CS-UCNP having the positive ζ-potential (12–30 mV). The CS-UCNP@Ale-P(DMA-AMPS) were not significantly internalized by the carcinoma cells due to negatively charged cell membranes that prevented the mutual contacts with particles. It can be thus concluded that the Ale-P(DMA-AEC)-DY-615-coated CS-UCNP showed a favorable cellular uptake that makes them a suitable candidate for cell labeling and prospectively for PDT of various tumors.

## Supplementary Information

Supplementary Information.

## Data Availability

The raw data required to reproduce these findings are available at the first author: oleksa@imc.cas.cz. The processed data required to reproduce these findings are available to download from oleksa@imc.cas.cz.
