# Supplementary material for: Poly(N,N-dimethylacrylamide)-coated upconverting NaYF4:Yb,Er@NaYF4:Nd core–shell nanoparticles for fluorescent labeling of carcinoma cells
Source: Sci Rep. 2021 Nov 1;11:21373. doi: 10.1038/s41598-021-00845-y (PMC8560758; doi:10.1038/s41598-021-00845-y)
Supplement: Supplementary file 1 — Supplementary Information. [file 41598_2021_845_MOESM1_ESM.docx]

**SUPPORTING INFORMATION**


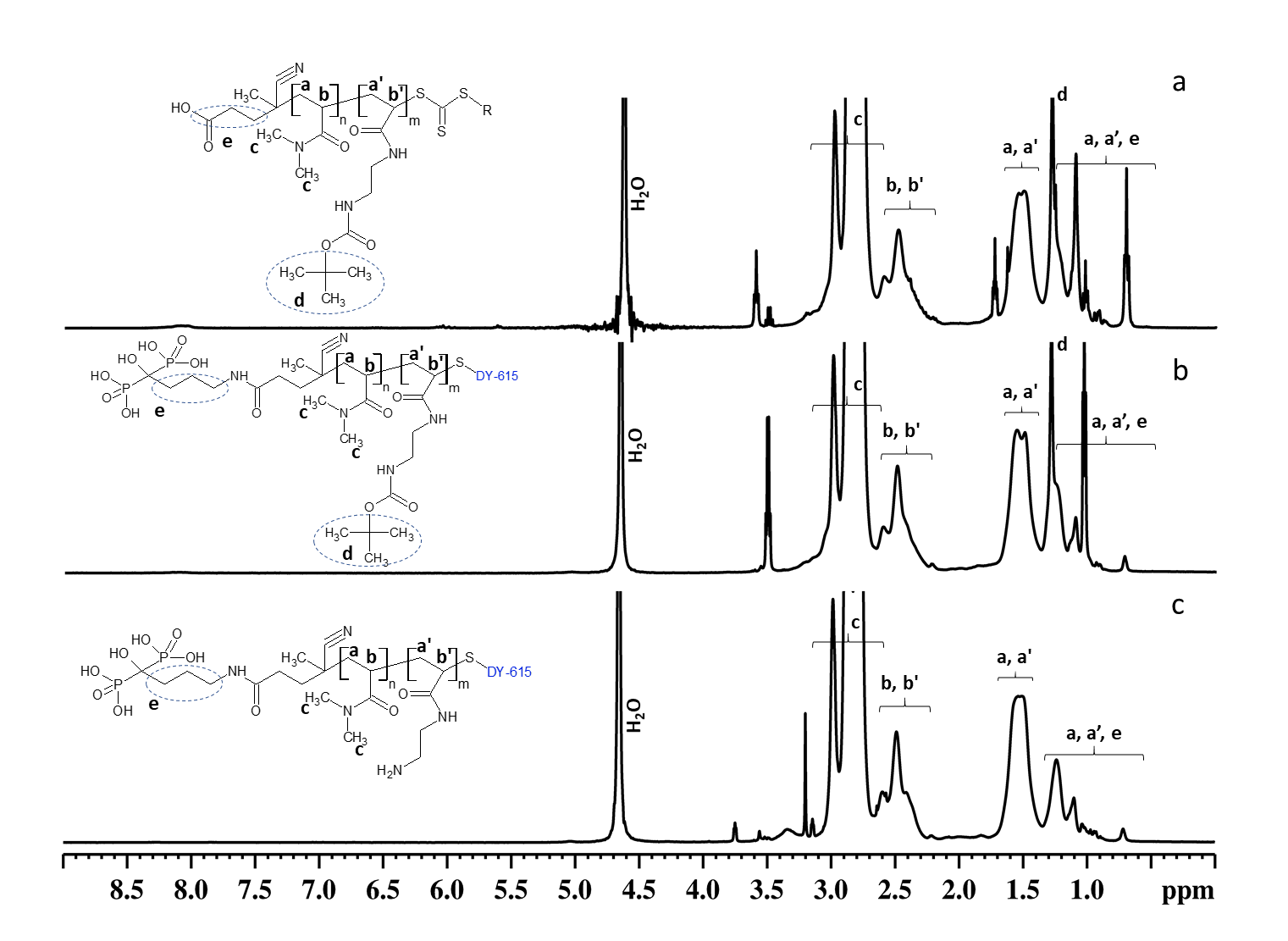


Figure S1. ^1^H NMR spectra of (a) P(DMA-AEC-Boc), (b) Ale-P(DMA-AEC-Boc)-DY-615, and (c) Ale-P(DMA-AEC)-DY-615 copolymers.


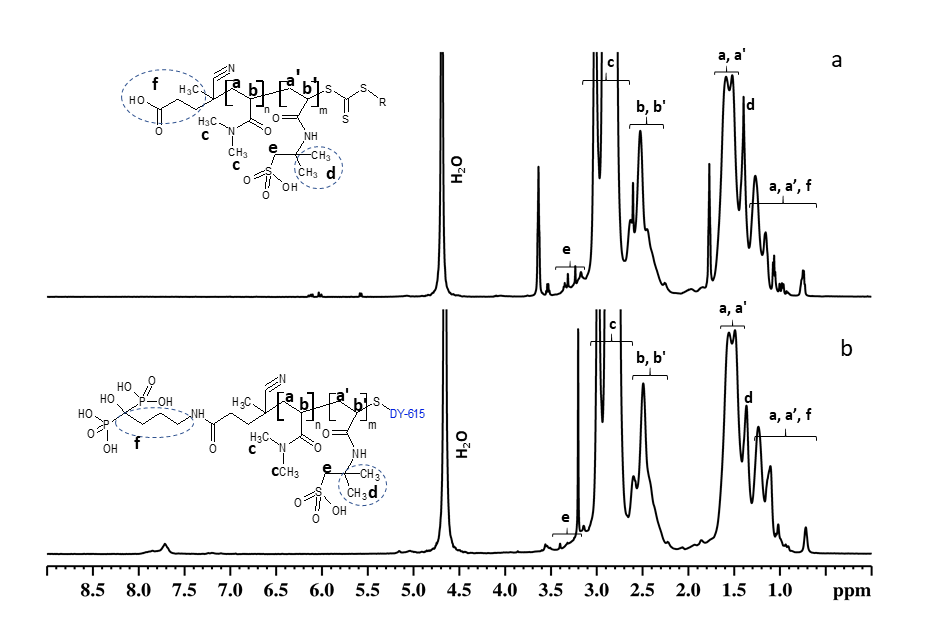


Figure S2. ^1^H NMR spectra of (a) P(DMA-AMPS) and (b) Ale-P(DMA-AMPS)-DY-615 copolymers.


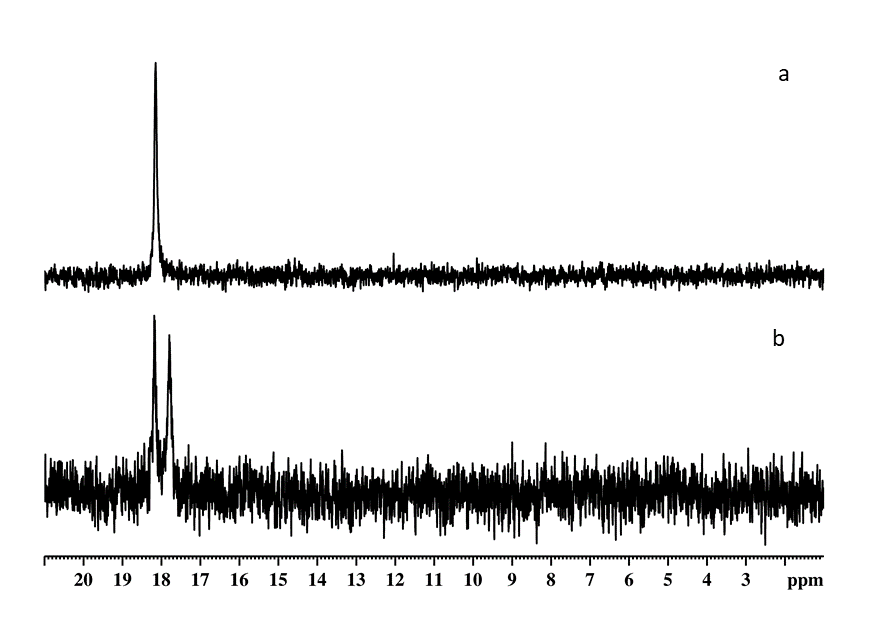


Figure S3. ^31^P NMR spectra of (a) Ale-P(DMA-AEC-Boc)-DY-615 and (b) Ale-P(DMA-AMPS)-DY-615 copolymers.

Figure S4. XRD diffractograms of C-UCNP (blue) and CS-UCNP (red).

|  |  |
| --- | --- |

Figure S5. Photoluminescence upconversion spectra of C-UCNP (red) and CS-UCNP (blue) nanoparticles (1.5 mg/ml) in (a) hexane and (b) water at 980 nm excitation. C-UCNP - NaYF_4_:Yb,Er; CS-UCNP - NaYF_4_:Yb,Er@NaYF_4_:Nd.

Figure S6. (a) UV-Vis absorption spectra of neat CS-UCNPs and CS-UCNPs coated with Ner-PEG, Ale-P(DMA-AEC)-DY-615, Ale-P(DMA-AMPS)-DY-615, and Ale-PDMA-DY-615. (b) Normalized photoluminescence excitation (dash; λ_em_ 637 nm) and emission (solid; λ_ex_ 621 nm) spectra of Ale-P(DMA-AEC)-DY-615- (blue), Ale-P(DMA-AMPS)-DY-615- (green), and Ale-PDMA-coated CS-UCNP (black) particles.

Figure S7. Photoluminescence upconversion spectra of Ale-P(DMA-AEC)-DY-615- (red), Ale-P(DMA-AMPS)-DY-615- (blue), and Ale-PDMA-DY-615-coated CS-UCNP (black) particles excited with 140 fs pulsed laser at (a) 808 and (b) 980 nm.


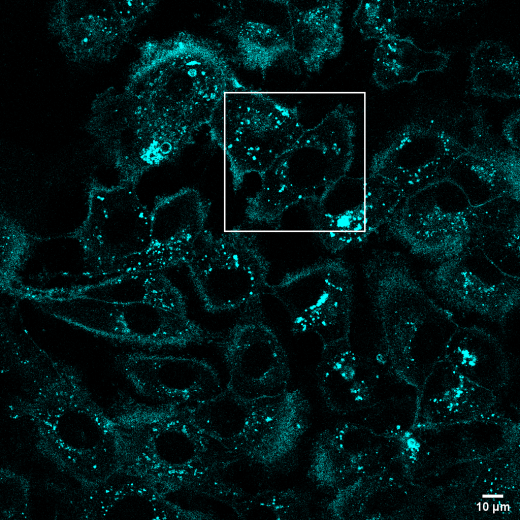

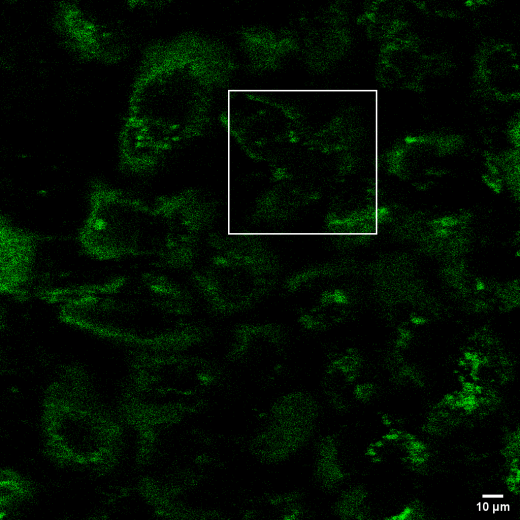

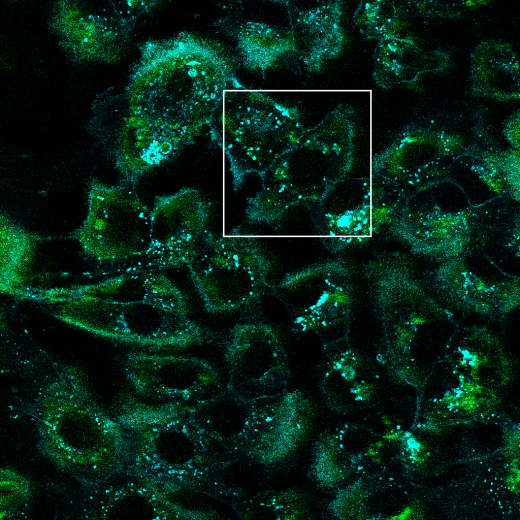

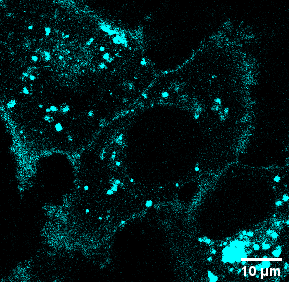

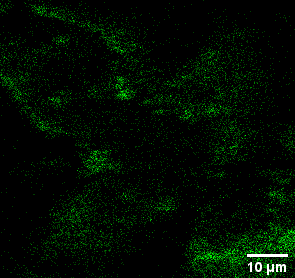

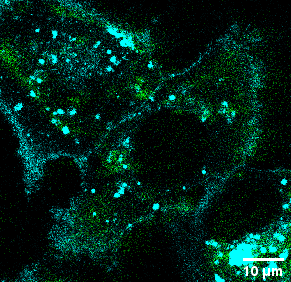


a

b

c

d

e

f

Figure S8. Distribution of CS-UCNP@Ale-P(DMA-AEC)-DY-615 nanoparticles in hepatocellular carcinoma cells at 980 nm excitation (laser power of 30–50 mW). (a, d) Cell membranes (blue) were stained with CellMask^TM^ green and (b, e) nanoparticles were green; (c, f) overlay of (a, b) and (d, e); (d, e, f) detailed section of (a, b, c). CS-UCNP - NaYF_4_:Yb,Er@NaYF_4_:Nd.
